# Primary cutaneous infections with non-tuberculous mycobacteria: a report of 6 cases

**DOI:** 10.1186/s12879-024-10134-4

**Published:** 2024-11-02

**Authors:** Qi-Hao Yao, Hui-Lin Zhi, Xiu-Jiao Xia, Ze-Hu Liu

**Affiliations:** 1 Department of Dermatology, Hangzhou Third People’s Hospital, Zhejiang University School of Medicine, Hangzhou, Zhejiang 310009 China; 2 Zhejiang University School of Medicine, Hangzhou, China

**Keywords:** Metagenomic next-generation sequencing, Skin and soft tissue infection, Non-tuberculous mycobacterium, Case-series study

## Abstract

**Background:**

The incidence of non-tuberculous mycobacterium infection has shown a gradual increasing trend in recent years, among which cutaneous manifestations as an important aspect. This study aimed to describe the clinical features and microbiological findings in 6 cases of primary cutaneous nontuberculous mycobacterium infection.

**Methods:**

In this retrospective study from June 2021 to June 2022, the clinical data and microbiological results of six cases diagnosed with primary cutaneous non-tuberculous mycobacterium infection in department of dermatology, Hangzhou Third People’s Hospital were analyzed.

**Results:**

All six cases were primary cutaneous non-tuberculous mycobacterium infections, four of which had a history of trauma or exposure, and two had an underlying disease that could lead to compromised immunity. All patients presented with erythema nodular skin lesions, four on the upper or lower extremities, one on the face, and one on the right hip. The histopathological findings of five patients who underwent biopsy were granulomatous inflammatory changes with mixed infiltration. Laboratory cultures using tissue or tissue fluid were all successful, including four *Mycobacterium marinum*, one *Mycobacterium abscessus*, and one *Mycobacterium avium*. Metagenomics next-generation sequencing detected results consistent with culture colonies in only two cases. With the exception of case 4, all patients responded well to oral medication, with a course of treatment ranging from 4 months to 1 year, and the prognosis was good.

**Conclusions:**

The clinical features of primary cutaneous non-tuberculous mycobacterium infection are often lacking in specificity, and the identification of related strains is difficult for a variety of reasons. Although the results of metagenomics next-generation sequencing are useful for pathogen spectrum identification, its diagnostic value should be carefully reevaluated under certain circumstances. Patients with suspected triggers who do not respond well to conventional treatments should be suspected as atypical infection and potential immunosuppression. If diagnosed and treated promptly, the prognosis of primary cutaneous non-tuberculous mycobacterium infection is generally good.

## Background

The genus *Mycobacterium* comprises a diverse group of microorganisms found extensively in the natural environment, including well-known pathogens like *Mycobacterium tuberculosis* and *Mycobacterium leprae*, as well as atypical ones as *Mycobacterium abscessus*, *Mycobacterium avium complex*, and *Mycobacterium marinum* [1]. The incidence of Mycobacterial infections has risen in recent years, a trend attributed to advancements in laboratory testing capabilities and the widespread adoption of epidemiological studies [2]. However, the diverse clinical presentations, specificity of affected sites, and the potential for delayed or inappropriate treatment can sometimes render the standard diagnostic process challenging [3–5].

The traditional gold standard for diagnosis, laboratory culture showed high specificity and the ability to isolate strains for subsequent susceptibility testing and identification [6]. However, being time-consuming and less sensitive, this method struggles to detect and identify slow-growing or hard-to-culture strains, thus potentially delaying diagnosis and treatment [7]. On the other side, advances in microbiological detection technologies, such as metagenomics next-generation sequencing (mNGS), matrix-assisted laser desorption ionization time-of-flight mass spectrometry (MALDI-TOF MS), and sequencing of 16 S rRNA and the internal transcribed spacer (ITS), have enabled the detection of multiple pathogens with enhanced sensitivity and expedited speed.

We reviewed six cases of non-tuberculous mycobacteria (NTM) infection that used both traditional culture and mNGS for pathogens identification, and compared their differences in clinical features and presentation (Table 1).

**Table 1:** Information and the results of microbiological examination about the 6 cases

Patient	Age/Gender	Site/distribution	Trigger/underlying disease	Strain identification by MALDI-TOF/NGS	MALDI-TOF Scores	Culture/NGS results	Histopathology	Treatment	Duration
1	48/F	Right hip /Localized	None/SLE	*M. avium*	N/A	Positive/Positive	Granuloma	Azithromycin 0.5 g/d, rifampicin 0.45 g/d, ethambutol hydrochloride 1 g/d	5 months
2	64/F	Right lower extremity/ sporotrichoid	Trauma/ Postoperative thyroid tumor	*M. marinum*	1.969	Positive/Positive	Granuloma	Rifampin 0.6 g/d, clarithromycin 0.25 g 2/d	4 months
3	39/F	Left thumb/Localized	None/None	*M. marinum*	2.2	Positive/Negative	Granuloma	Doxycycline 0.1 g 2/d	12 months
4	27/F	Right face/ Scattered	Cosmetic injection/None	*M. abscessus*	1.806	Positive/Negative	N/A	None	Loss to follow-up
5	49/F	Left upper extremity/ sporotrichoid	Trauma related to fishing /None	*M. marinum*	2.034	Positive/Negative	Granuloma	Rifampin 0.45 g/d, clarithromycin 0.25 g 2/d	9 months
6	37/M	Right upper extremity/ sporotrichoid	Tropical fish tank cleaning/None	*M. marinum*	2.079	Positive/Negative	Granuloma	Rifampin 0.45 g/d, clarithromycin 0.25 g 2/d	8 months

N/A in MALDI-TOF Scores: The strain died during subsequent transferring, and the specific MALDI-TOF score was no longer available after the preservation period

## Methods

This is a retrospective study from June 2021 to June 2022. Patients of primary cutaneous NTM infection who went through the mNGS examinations for further support was enrolled at Department of Dermatology, Hangzhou Third People’s Hospital. The inclusion criteria of selected cases are as follows:1) ≥ 18 years old; 2)The infection was limited to skin and subcutaneous tissues, with no disseminated manifestations, significant respiratory or other systemic discomfort. Clinical data, morphological characteristics of lesions and results of microbiological examination were documented for comparison. Informed consent to participate was obtained from all of the participants in the study in the follow-up.

Microbiological tests include direct smear acid-fast staining, culture for fungi and mycobacteria, and mNGS. Mycobacterial culture of NTM was performed on a modified Lowenstein–Jensen medium (Hangzhou Genesis Biodetection & Biocontrol Co., Ltd. GENESIS, China) at 25℃. The suspected colonies were further identified by MALDI-TOF MS. The specific scores are shown in Table 1.

The diagnosis and strain identification were based on traditional culture and subsequent MALDI-TOF MS, with the support of mNGS.

## Results

### The patient’s clinical condition

#### Case 1

**Fig. 1:**
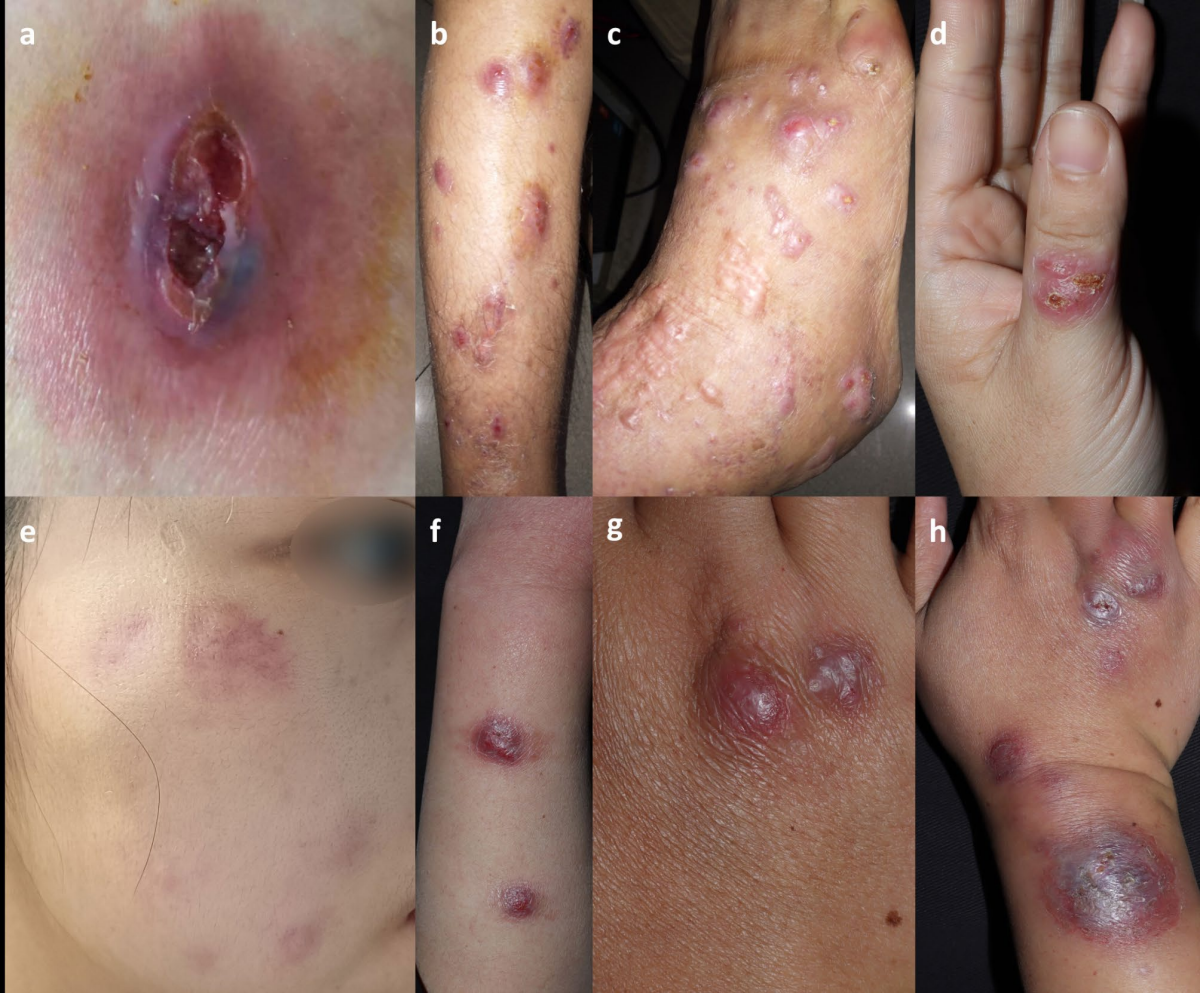
The Clinical pictures of the patients. a: A single Erythematous nodule and superficial ulceration with exudate and edema in the right hip in case 1. b, c: Sporotrichoid erythematous nodular lesions on the right lower extremity, including the right foot, right ankle and the front of the calf in case 2. d: Localized erythema papules covered with yellow crusts on the left thumb in case 3. e: Scattered erythema plaques on the right face in case 4. f: Sporotrichoid nodules on the left forearm in case 5. g: The initial nodules on the dorsal right hand in case 6. h: After oral doxycycline for 1 month, the lesion progressed to the right forearm, with ulceration and scarring in case 6

was a 48-year-old woman who had erythematous and abscess for 4 months. Her lesions showed itch and hurt. Because of her systemic lupus erythematosus (SLE) more than 20 years, she received oral prednisone 10 mg/d, azathioprine 50 mg/12 h, hydroxychloroquine 200 mg/12 h for the recent half a year. The patient had stable SLE control and no trauma before the disease. A localized erythematous nodule and painful superficial ulceration about 4 cm×5 cm companied by exudate were observed in the patient’s left hip with edema (Fig. 1a).

#### Case 2

was a 64-year-old woman presenting with erythematous nodular lesions on the right lower extremity. The lesions began with ankle trauma 2 years ago and gradually became multiple ones in the right lower limb over a period of 2 years (Fig. 1b and c). Oral antifungal drugs in the first few months in local hospital showed no effect. She had a thyroid tumor surgery several years ago.

#### Case 3

was a 39-year-old woman with an isolated lesion on the left thumb for 2 months. The patient reported no clear trigger before the disease, and the initial lesions presented as blisters, which later formed erythema papules covered with yellow crusts (Fig. 1d).

#### Case 4

was a 27-year-old female who presented with scattered erythema plaques on the face for 6 months (Fig. 1e). The patient had received cosmetic injections on the lesion several times prior to onset.

#### Case 5

is a 49-year-old female fisherman who presented with nodules on her left forearm after trauma related to fishing. The lesion did not develop significantly in the course of more than 1 year without treatment (Fig. 1f).

#### Case 6

was a 37-year-old man with erythema nodules on the dorsal right hand for 10 days (Fig. 1g). The patient took doxycycline orally for 1 month, and the lesion progressed to the right forearm, with ulceration and scarring (Fig. 1h). The patient reported regularly cleaning the fish tank at home. The fish species raised in the tank are *Pterophyllum* genus.

### Histopathology and microbiological results of the patients

**Fig. 2:**
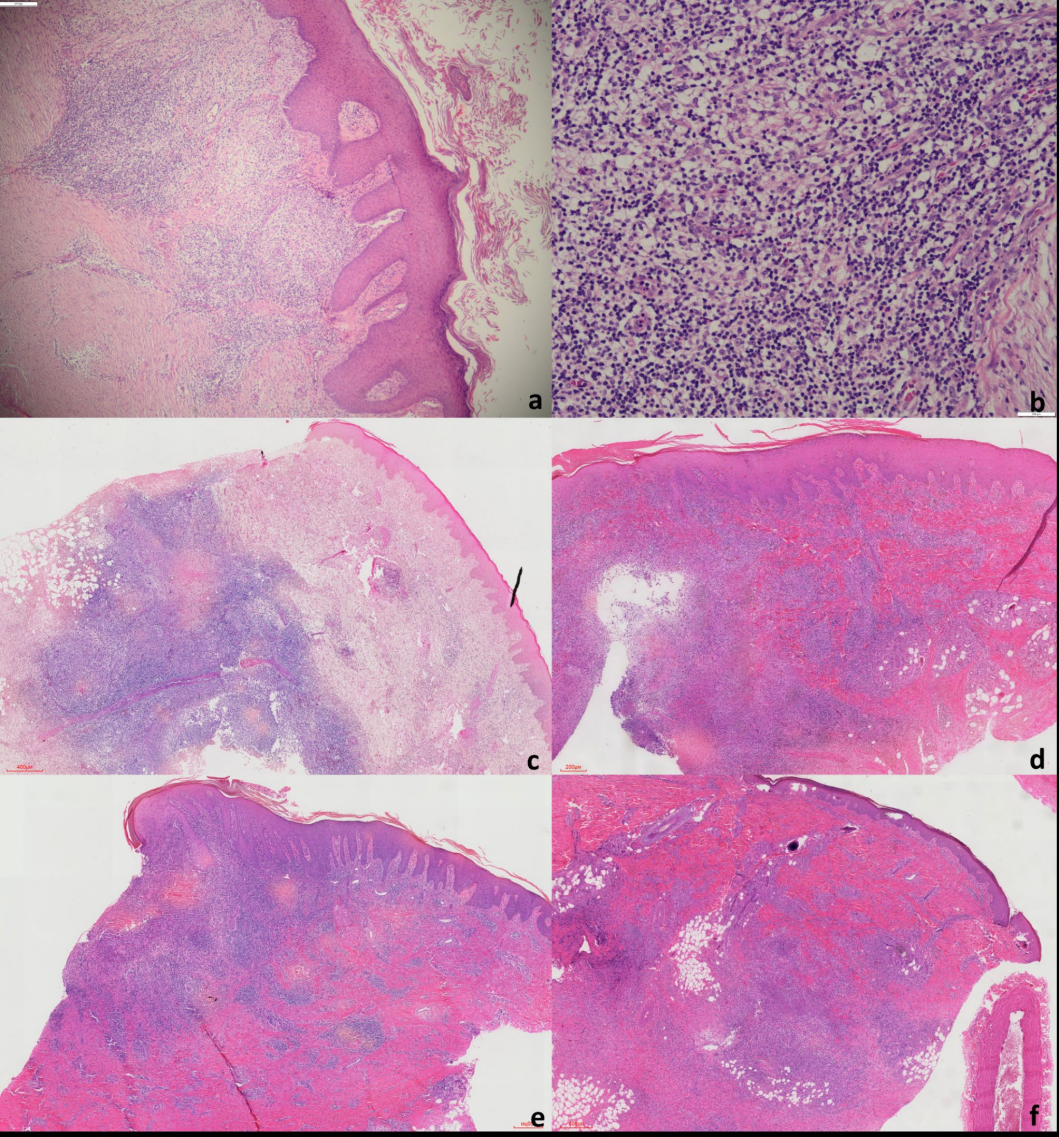
The histopathological results of the lesions’ biopsy, stained in hematoxylin-eosin. a: Granulomatous inflammation in case 1. (×40). b: Mixed infiltration of inflammatory cells including histiocytes, lymphocytes, neutrophils and multinucleated giant cells in case 1. (×200). c, d, e, f: Granulomatous inflammation in case 2,3,5,6, respectively (The corresponding unit length has been indicated in each figure)

Except for case 4, skin biopsies were performed for histopathology and microbiological examinations. Case 4 refused biopsy, whose tissue fluid was sent for examinations, and was not followed up. The histopathology results were shown in Table 1, in hematoxylin-eosin (HE) staining (Fig. 2a, c, d, e and f), with an infiltration of mixed inflammatory cells including histiocytes, lymphocytes, neutrophils and multinucleated giant cells at high power (Fig. 2b). Necrotic areas could be seen in the center of some granulomatous structures. Ziehl–Neelsen and Periodic Acid-Schiff (PAS) staining failed to found pathogens.

**Fig. 3:**
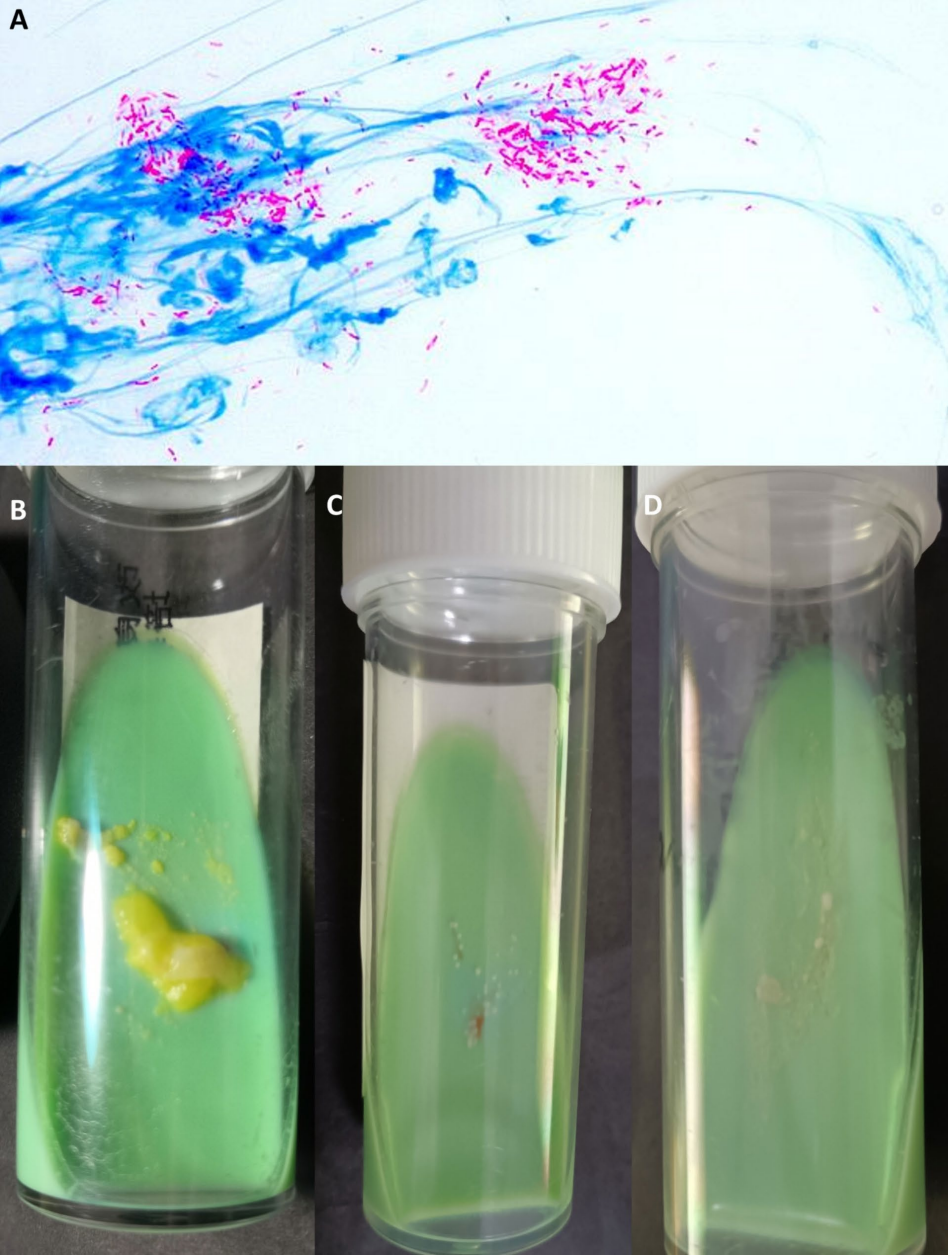
Pictures of Ziehl–Neelsen staining and culture of mycobacteria. a: Ziehl–Neelsen staining of the smear in case 1, showing multiple acid-fast bacilli. b: *Mycobacterium avium* from case 1, as large yellow colonies grown on modified Lowenstein–Jensen medium. c: *Mycobacterium abscessus* from case 4, as small and scattered cream-colored colonies on modified L-J medium. d: *Mycobacterium marinum* from case 6, as yellowish colonies on modified L-J medium

Ziehl–Neelsen stain of the smear showed acid fast bacilli in case 1 with negative results in others (Fig. 3a). In all cases, non-tuberculous mycobacteria were cultured successfully and identified using MALDI-TOF MS as 1 *Mycobacterium avium* in case 1 (Fig. 3b), 1 *Mycobacterium Abscessus* in case 4 (Fig. 3c), and 4 *Mycobacterium marinum* in rest patients (Fig. 3d). mNGS were used to further determine the presence and detailed species of mycobacteria in the case. The results of cases 1 and 2 confirmed the presence of related pathogens with the culture results, as *M. avium* and *M. marinum*, respectively. However, no consistent mycobacterium was found in the other four patients.

### Treatment and outcome

Except case 4, all patients were treated in our hospital and recovered. The specific treatment, dosage and duration are shown in Table 1. In case 6, lesions were still progressing with oral doxycycline for 1 month, but improved after changing drugs. All the treated patients had no recurrence or disseminated manifestations at follow-up, with only scars or hyperpigmentation remained.

## Discussion

Extrapulmonary tuberculosis, with a notable increase in cutaneous cases, has garnered heightened attention in clinical practice in recent years. Owing to enhanced clinical comprehension and refined diagnostic techniques, there has been a marked improvement in the detection rate [2]. Notably, even among patients with infections caused by NTM, such as *Mycobacterium avium* complex (MAC), cutaneous manifestations constitute a significant aspect [3]. However, case reports about primary cutaneous infections of NTM are still relatively rare.

Cutaneous infection of NTM is usually secondary to a history of trauma or exposure associated with a specific strain. For example, *Mycobacterium marinum* is associated with aquatic exposure [8]. While rapidly growing mycobacteria, including *Mycobacterium abscessus*,* Mycobacterium fortuitum* and *Mycobacterium chelonae*, could be associated with surgery, cosmetic injections, prosthesis implants, and catheters [9–11]. In particular, because some mycobacteria as MAC and *Mycobacterium haemophilum* exist in the natural environment of soil or water, contact with these potential infectious agents can sometimes cause infection [12].

At the same time, NTM is one of the important causes of nodular lymphangitis (Sporotrichoid Lymphocutaneous Infections), among which *M. marinum* is the most common [13]. According to literature review, more than half of the patients presented with isolated lesions, while 25% of the patients may presented with sporotrichoid distribution [14]. Lesions could be banded along lymphatic vessels but do not always reach the regional lymph nodes [15]. The lesions often lack specificity in morphology, and may present as papulonodular, erythematous or ulcerative ones. Some patients present with deep infection of subcutaneous tissue, affecting tendon, joint and bone marrows, but it is relatively rare [16].

Immunosuppression caused by malignancy or the use of oral glucocorticoids may greatly increase the incidence of NTM infection or alter the general course of the disease, leading to rapid progression [17]. In 5 out of 6 of our cases, patients had risk factors associated with NTM infection, including trauma or an underlying disease that could lead to compromised immunity. In general, the presence of atypical pathogens, including NTM and other rare ones, should be suspected in patients with probable triggers (trauma/occupational-related/iatrogenic injury) who do not respond well to conventional treatment. A variety of drugs, including clarithromycin, ethambutol, rifampicin, doxycycline, etc., can be used in the treatment of NTM cutaneous infection [18].

However, diagnosing and identifying the pathogen using traditional methods remains a question. The difficulty primarily came from the need for specific culture conditions, the slow growth rate of certain strains, and the low pathogen load within lesions [3]. Furthermore, pre-hospital topical debridement or the administration of antibiotics complicates detection in some patients by reducing the pathogen burden [19]. If it is lucky enough to culture or detect a specific pathogen, it is sometimes difficult to tell which member of the complex it belongs to even using MALDI-TOF or 16s RNA sequencing. These factors combined brings some difficulties to clinical diagnosis.

In recent years, the benefits of emerging mNGS have been underscored, particularly their high detection rates against low abundance and rare pathogens across various sampling sites and sample types [20, 21]. The results of mNGS were interpreted differently in different sites: in sites traditionally considered sterile as the central nervous system or the lower respiratory tract, limited reads could support the diagnosis. However, there is a lack of uniform standards or expert consensus when it comes to bacterial colonization sites such as skin [22, 23]. Interpretation of results from these sites should depend on joint evaluation and analysis by physicians and sequencing institutions [24].

A study focusing on skin and soft tissue infection (SSTI) demonstrated that mNGS is more efficient than traditional cultures, particularly in the sensitivities of detecting rare or mixed pathogens [25]. Due to the special culture conditions or the low survival ability of these rare pathogens, mNGS could show better sensitivity and detection rate than conventional culture. For a specific patient sample, mNGS provides an unbiased and comprehensive screening in pathogen spectrums. This helps doctors conduct an initial assessment, especially when the cause remained unclear. However, in certain cases, just as what our cases indicated, the results of mNGS might not be consistent with the actual situation and could not meet the needs of diagnosis. Its ability to detect and identify NTM in SSTI should not be overestimated. Of the six cases, negative culture and mNGS results were found in zero and four cases, respectively. This may be due to the fact that other colonizing bacteria on the skin interfered with the detect of NTM sequences, or it may be due to the low NTM load and the characteristics of bacterial structure. However, it is satisfactory that the responsible mycobacteria detected by mNGS are consistent with those detected by MALDI-TOF MS in culture, suggesting that once mNGS has a positive result, it can be used as a good testimony to traditional testing methods.

Because of the complexity of mNGS results, the pathogens detected may cause interference in clinical diagnosis. The unsatisfactory results of mNGS could be attributed to a combination of factors, including the initial sample processing, the sequencing depth, and reliance on the database. The following optimization directions may enhance the value of mNGS as a diagnostic approach for SSTI. Prior to the decision to use mNGS, appropriate sampling sites and suspected pathogens should be identified in conjunction with traditional diagnostic methods, clinical symptoms, and imaging findings. Deep dermis, subcutaneous tissue or deep pus and tissue fluid should be used during sample collection to avoid the interference of superficial colonizing bacteria for sequencing. Standardizing the storage, transport and sequencing process could potentially reduce the loss of information. Finally, the construction of database should be strengthened continuously to provide technical support. In conclusion, mNGS should always be considered as a complementary tool to traditional diagnostic methods rather than a replacement.

There are still some limitations of our study. The patients are from the same region, and the sample size is not big enough, leading to the lack of generalizability of relevant conclusions. Patients with NTM infection in other regions should be evaluated by combining multiple methods to obtain more accurate conclusions about the meaning of mNGS. While mNGS has showed its value, it puts a certain demand on cost and technological platform, which leads to its limited application in specific groups of people [26]. This also limited the scope of our study.

Traditional laboratory culture, serving as an essential preliminary step, auxiliary evidence, and follow-up reference for molecular medical diagnostics and drug sensitivity testing, especially when there is a poor response to conventional anti-mycobacterial therapy, warrants renewed attention [27]. Although mNGS has shown special value in the diagnosis of SSTI, it should not be used as a primary diagnostic method or ‘golden standard’. In specific isolated cases, the role of mNGS may be more to provide a comprehensive assessment of pathogens to guide specific tests, especially when cultures fail or conditions are inadequate.

## Conclusion

In many countries, the knowledge and treatment of cutaneous NTM infections remained a problem. Medical personnel should not overestimate the value mNGS in small patient populations, and pay more attention to the routine culture, especially the choice of temperature and medium type. The main purpose of our report is to summarize the 6 cases using mNGS as diagnostic means, and to improve practitioners’ understanding of the diagnostic methods of this disease.

## Data Availability

No new gene sequences were generated after performing mNGS. For the raw data of mNGS, the corresponding author Ze-Hu Liu could be contacted, whose email as zehuliu@yahoo.com.Other Data is provided within the manuscript.

## Abbreviations

mNGS: metagenomics next-generation sequencing
MALDI-TOF MS: matrix-assisted laser desorption ionization time-of-flight mass spectrometry
ITS: internal transcribed spacer
NTM: non-tuberculous mycobacteria
SLE: systemic lupus erythematosus
HE: hematoxylin-eosin
MAC: Mycobacterium avium complex
SSTI: skin and soft tissue infection
